# Construction of Metal Organic Framework-Derived Fe-N-C Oxidase Nanozyme for Rapid and Sensitive Detection of Alkaline Phosphatase

**DOI:** 10.3390/nano13182496

**Published:** 2023-09-05

**Authors:** Mengmeng Pan, Ming Wang, Linjiao Yang, Yongli Song, Ming Jiang, Xu Yu, Li Xu

**Affiliations:** 1 Tongji School of Pharmacy, Huazhong University of Science and Technology, Wuhan 430030, China; d202081590@hust.edu.cn (M.P.); d202081597@hust.edu.cn (Y.S.); 2001020636@hust.edu.cn (M.J.); 2 Department of Clinical Laboratory, Renmin Hospital of Wuhan University, Wuhan 430060, China; morgan@whu.edu.cn; 3 Hubei Jiangxia Laboratory, Wuhan 430200, China

**Keywords:** metal-organic framework, nanozyme, oxidase-like enzyme, alkaline phosphatase, visual detection

## Abstract

Alkaline phosphatase (ALP) is a phosphomonoester hydrolase and serves as a biomarker in various diseases. However, current detection methods for ALP rely on bulky instruments, extended time, and complex operations, which are particularly challenging in resource-limited regions. Herein, we synthesized a MOF-derived Fe-N-C nanozyme to create biosensors for the coulometric and visual detection of ALP. Specifically, we found the Fe-N-C nanozyme can efficiently oxidize 3,3′,5,5′-tetramethylbenzidine (TMB) to generate blue-colored tetramethyl benzidine (TMB_ox_) without the need for H_2_O_2_. To construct the biosensor, we incorporated the ALP enzymatic catalytic reaction to inhibit the oxidation of TMB by Fe-N-C oxidase nanozyme. This biosensor showed rapid and highly sensitive detection of ALP in both buffer and clinical samples. The limit of detection (LOD) of our approach could be achieved at 3.38 U L^−1^, and the linear range was from 5 to 60 U L^−1^. Moreover, we also developed a visual detection for ALP by using a smartphone-based assay and facilitated practical and accessible point-and-care testing (POCT) in resource-limited areas. The visual detection method also achieved a similar LOD of 2.12 U L^−1^ and a linear range of 5–60 U L^−1^. Our approach presents potential applications for other biomarker detections by using ALP-based ELISA methods.

## 1. Introduction

Alkaline phosphatase (ALP) is a kind of phosphomonoester hydrolase that is ubiquitously present in the human body and closely reflects the health state of patients. It has proven valuable in the clinical diagnosis of a variety of diseases, including liver disease, secondary liver cancer, rickets, and prostate cancer [1,2]. At present, several methods, such as electrochemistry, fluorescence, or chromatography, have been utilized for ALP detection [3,4,5,6,7]. However, these analytical methods usually rely on bulky precision instruments, extended detection times, and complex operation steps. Meanwhile, in some resource-limited regions, a more practical and visually accessible point-of-care testing (POCT) method for ALP detection was preferred. Therefore, it is urgent to develop simple, rapid, and sensitive methods for ALP detection to address the aforementioned challenges.

Recently, researchers have demonstrated the feasibility of the construction of a POCT method for ALP detection by using a real-time colorimetric change caused by enzyme catalysis [8,9,10,11,12]. However, the natural enzymes were difficult to obtain and store at a high cost. To solve the problems, nanozymes were developed to mimic nature’s enzymes and perform these functions. Nanozymes were a kind of artificial nanomaterial that could simulate the catalytic activity of natural enzymes [13,14]. Due to their advantages of high stability, easy synthesis, tunable catalytic activity, and minimal requirement for special enzyme catalytic environments, they have attracted intensive attention from scientists [15,16,17,18]. Up to now, various nanomaterials, such as inorganic materials, metal oxides, and metal-organic frameworks (MOFs), have been exploited to simulate one or more enzyme activities and have been successfully applied in many fields, such as biosensing, chemical synthesis, pollutant removal, and disease treatment [19,20,21,22,23]. However, the heterogeneous catalytic nature of most nanozymes and the morphologies, sizes, and surfaces of the materials could have significant impacts on catalytic activity, resulting in unpredictable catalytic outcomes [24,25]. As a kind of nanomaterial with a uniform porous structure, MOF possessed the possibility of design and modification at the molecule level, enabling the construction of diverse nanozymes. For example, researchers have reported the synthesis of MOF-derived nanozymes for detecting prostate-specific antigens or engineering MOF structures to achieve catalytic synergistic antibacterial therapy [24,26]. Taking advantage of MOF, this study endeavored to synthesize MOF-derived nanozymes with a straightforward process, controllable enzyme activity, and exceptional catalytic activity for ALP detection.

**Scheme 1:**
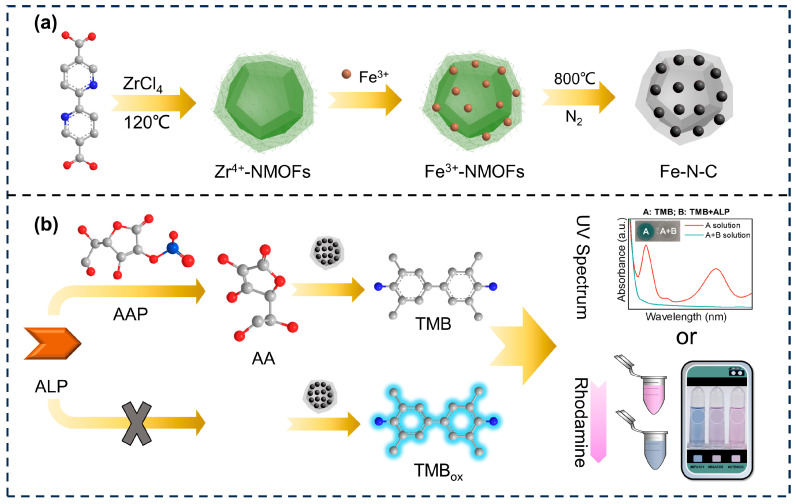
Schematic of the detection process of ALP. (**a**) the preparation of Fe-N-C. (**b**) the colorimetric and naked-eye detection principle of ALP.

Based on our previous reports, the bipyridine ligand of UIO-67 exhibited the capability to chelate metal ions onto the MOFs and evenly distribute them within the skeleton structure [27,28]. As shown in Scheme 1a, we first achieved uniform modification of Fe^3+^ ions within the Zr^4+^-NMOFs structure through the chelation of Fe^3+^ ions with a bipyridine ligand. Subsequently, the Fe^3+^-NMOFs nanomaterials were calcined in a N_2_ atmosphere, resulting in the formation of Fe-N-C nanozymes with a secondary structure. The interaction between the tiny nanoparticles and the appropriate surface-active sites facilitated the enzyme activity, making it exert better nanozyme properties. Moreover, unlike nanozymes with peroxidase (POD) activity, we found that the MOF-derived Fe-N-C nanozyme exhibited high oxidase (OXD)-mimic catalytic activity, enabling the oxidation of O_2_ to generate an abundance of reactive oxygen species (ROS, O_2_^•−^) without requiring H_2_O_2_. Due to the good OXD-like activity of the Fe-N-C nanozyme, we are supposed to construct a biosensor to detect the ALP with the enzymatic catalytic reaction, such as inhibiting the oxidation of the TMB.

In previous reports [29,30], the ALP could hydrolyze the phosphate substrate, 2-phospho-L-ascorbic acid (AAP), to produce ascorbic acid (AA). Then the AA reduced the Ag^+^ in the AuNPs solution or the Cu^2+^ ions in a polyT-DNA solution, resulting in the formation of AuNPs@Ag or fluorescence Cu clusters. These systems allowed for colorimetric or fluorescence-based detection of nucleic acids and proteins. Inspired by these, we designed a biosensor for ALP detection by using the produced AA to inhibit the oxidation of TMB by Fe-N-C oxidase nanozyme. This feature allowed the system to be utilized for the rapid and sensitive detection of ALP, as depicted in Scheme 1b. In this detection system, the level of ALP activity is reflected by measuring the extent of the oxidation reaction (e.g., the oxidation degree of TMB). In addition, in order to meet the POCT requirement, we performed the visual detection by measuring the RGB values of the solution. The images were taken by a smartphone and processed through an imaging APP, achieving the purpose of colorimetric quantitative detection of ALP.

In summary, we have successfully synthesized a MOF-derived nanozyme, Fe-N-C nanozyme, which exhibits exceptional OXD-mimic activity. By incorporating the ALP enzyme catalytic reaction, we constructed a biosensor for rapid and highly sensitive detection of ALP by inhibiting the oxidation of TMB by Fe-N-C nanozyme. Notably, our method offered the advantage of not requiring the addition of H_2_O_2_ in the detection system, making our approach simple and stable. Meanwhile, the visual detection of our method facilitated the POCT detection of ALP in resource-limited areas. In the future, this strategy might be extended to detect other biomarkers with ALP-based ELISA methods.

## 2. Materials and Methods

### 2.1. Chemicals and Regents

Ethanol, tetrahydrofuran (THF), N,N-dimethylformamide (DMF), ZrCl_4_, dimethyl sulfoxide (DMSO), methylene blue (MB), FeCl_3_·6H_2_O, benzoic acid (TA), NaCl, Na_2_HPO_4_·12H_2_O, NaH_2_PO_4_·2H_2_O, H_2_O_2_ and ascorbate magnesium phosphate (AAP) were purchased from Sino Chemical Reagent Co., Ltd. (Shanghai, China). 2,2′-bipyridine-5,5′ dicarboxylic acid (H_2_BPY), dihydroethidium (DHE), and TMB were obtained from Aladdin Reagent Co., Ltd. (Shanghai, China). ALP was purchased from Thermo Fisher Technology Co., Ltd. (Suzhou, China). Amylase (AMY), lignin peroxidase (LIP), choline oxidase (CHOx), catalase (CAT), glucose oxidase (GOX), trypsin (TRY), and cholesterol oxidase (CHOL) were purchased from McLean Biochemical Technology Co., Ltd. (Shanghai, China).

### 2.2. Apparatus

The field emission scanning electron microscope (SEM) (MIRA 3, TESCAN Brno, s.r.o., Brno-Kohoutovice, Czech Republic) was used to observe the morphology of Fe-N-C. Transmission electron microscope (TEM) images were obtained by the Talos F200S G2 field emission microscope (Thermo Fischer, Waltham, MA, USA). The surface area was measured by nitrogen adsorption on a TriStar II surface area analyzer (Micromeritics, Norcross, GA, USA). The chemical compositions of Fe-N-C nanoparticles were verified by XPS (Thermo Fischer, Waltham, MA, USA). Fluorescence spectra were taken by a fluorescence spectrophotometer (F-4600, Hitachi, Tokyo, Japan). The information on crystallization was inspected by X-ray diffraction spectra (Ultima IV, Rigaku, Tokyo, Japan).

### 2.3. Synthesis of Fe-N-C Nanozyme

A total of 244 mg ZrCl_4_ and 233 mg H_2_BPY were dispersed in 100 mL of DMF, respectively. After 5 min of ultrasonic treatment, these two solutions were mixed into 250 mL glass bottles with the addition of 4650 µL of acetic acid. The mixture was heated at 120 °C for 18 h. After cooling to room temperature, the white solids were collected by centrifugation at 9500 rpm, washed with DMF once, THF once, ethanol three times, and deionized water three times, respectively, giving the purified Zr^4+^-NMOFs, which were finally dispersed in ethanol. Next, the FeCl_3_ ethanol solution (0.1 M) was added to the Zr^4+^-NMOFs ethanol solution with 20 µmol of FeCl_3_ per mg of MOF, and the mixture was incubated overnight on a rotary vibrator at room temperature. The product was washed with ethanol and dried at 60 °C to obtain a yellow powder material, i.e., Fe^3+^-NMOFs. Then, Fe^3+^-NMOFs were calcinated in a N_2_ atmosphere at 800 °C for 2 h, and the Fe-N-C nanozyme was thus obtained.

### 2.4. Catalytic Kinetic Experiment of TMB

Fe-N-C nanozyme was incubated with TMB at different concentrations, and the UV absorption value was continuously monitored at a wavelength of 652 nm. The *K_m_* and *V_m_* constants were obtained by fitting the Michaelis-Menten equation, i.e., *V* = *V_m_* × *C*/(*K_m_* + *C*). Where *V* is the initial catalytic reaction rate; *C* is the initial concentration of TMB; *V_m_* represents the maximum catalytic reaction rate; and *K_m_* stands for Michaelis constant.

### 2.5. Identification of ROS in the Fe-N-C Nanozyme Catalyzed System

The DHE assay was used to detect O_2_^•−^ in reaction systems. H_2_O as a control group and different Fe-N-C nanozyme concentrations were added to an aqueous solution containing 100 μM DHE and reacted for 2 h under dark conditions. The fluorescence emission spectra of the reaction solution were recorded at λ_ex_ = 370 nm.

The presence of •OH in the Fe-N-C nanozyme reaction system was verified by MB and TA experiments. The MB water solution (10 µg mL^−1^), H_2_O_2_ (10 mM), and Fe-N-C nanozyme were mixed. After incubating for 24 h, the supernatant was collected by centrifugation and subjected to UV absorption spectrum measurement. Similarly, TA aqueous solution (0.5 mM), H_2_O_2_ (100 mM), and Fe-N-C nanozyme (40 ug mL^−1^) were mixed and incubated for 24 h. After centrifugation, the supernatant of the solution was collected, and the fluorescence emission spectrum was recorded at λ_ex_ = 315 nm.

### 2.6. Detection of ALP with the Fe-N-C Nanozyme-Based Method

AAP (500 μM) was added to a Tris-HCl (pH = 9, 50 mM) buffer solution containing ALP as the ALP reaction solution. After the reaction was incubated at 37 °C for 40 min, 100 μL of this solution was added to the HAc-NaAc (pH = 3500 mM) buffer solution containing TMB (0.05 mg mL^−1^, 100 μL), followed by the addition of 2 μL Fe-N-C (1 mg mL^−1^) aqueous dispersion solution. The absorbance value at 652 nm was measured using a UV-vis spectrometer after 10 min. Meanwhile, the visual detection was performed with a smart phone (Redmi K30, Xiaomi Corporation, Beijing, China) and a commercially available APP (Color Collect, WX Color Tech Inc., Fuzhou, China). The operation procedure and the relative details were described in the supporting information (Figure S9).

### 2.7. Evaluation of the Fe-N-C Nanozyme-Based Method for the Detection of ALP

ALP of different concentrations (5, 10, 20, 30, 40, and 60 U L^−1^) was added to the ALP reaction for determining the linear relationship. The limit of detection (LOD) was calculated by the standard deviation of the response in the absence of ALP (SD) and the slope of the linearity (S), i.e., LOD = 3.3 × SD/S. The recoveries were obtained by dividing the percent of the assay value of the Fe-N-C nanozyme system by the added amount of ALP. 0.01 mM of Zn^2+^, Cu^2+^, Fe^3+^, Ca^2+^, K^+^, Mg^2+^, BSA, GSH, or Lys were added to the ALP reaction solution, respectively, and reacted at 37 °C for 40 min. The 100 μL solution was mixed with TMB (0.05 mg mL^−1^, 100 μL) HAc-NaAc buffer solution (pH = 3100 mM), and then 2 μL Fe-N-C (1 mg mL^−1^) aqueous dispersion solution was added. The absorbance value at 652 nm was measured using a UV-vis spectrometer. AMY, LIP, CHOx, CAT, GOX, TRY, or CHOL were used to replace ALP for specific detection.

### 2.8. Detection of ALP in Clinical Samples

Human serum samples were obtained from Renming Hospital of Wuhan University and diluted 10 times with Tris-HCl (pH = 9, 50 mM) buffer solution for reducing matrix interference and further testing. The protocol was approved by the Ethics Committee of Renmin Hospital of Wuhan University (approval number: WDRY2020-K061). The serum sample without AAP was taken as a blank sample and compared with the experimental sample after adding AAP for calculating the ALP concentration in clinical samples. Then, the testing procedure followed that in Section 2.6: P-nitrophenyl phosphate (P-NPP) substrate with a 2-amino-2-methyl-1-propanol (AMP) buffer as the standard method to detect the activity of ALP. In this design, ALP catalyzes the conversion of p-NPP to p-nitrophenol (p-NP) within the AMP buffer. The introduction of Mg^2+^ and Zn^2+^ ions enhanced the absorbance of p-NP at 410 nm. The change in absorbance correlates with the activity of ALP.

## 3. Results and Discussion

### 3.1. Characterization of Fe-N-C Nanozyme

**Figure 1:**
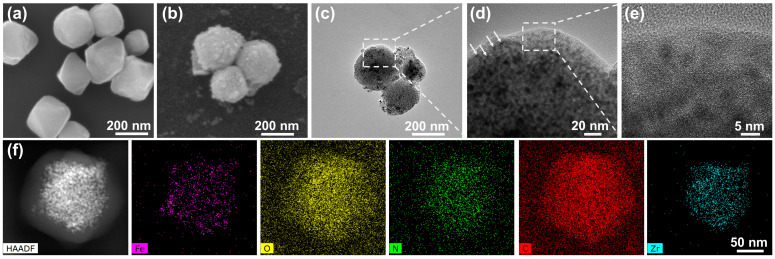
(**a**,**b**) SEM images of Zr^4+^-MOFs and Fe-N-C nanozymes, respectively. (**c**–**e**) TEM image and in the magnified view of Fe-N-C nanozyme. (**f**) High-resolution TEM image of Fe-N-C nanozyme and the corresponding element mapping images of Fe, O, N, C, and Zr, respectively.

The synthesis of Fe-N-C nanozymes involved three processes, as follows: Firstly, ZrCl_4_ reacted with the bipyridine ligand, H_2_BPY, at 120 °C for 18 h to obtain the Zr^4+^-NMOFs according to the procedures of our previous reports [27,28]. As shown in Figure 1a, the SEM (MIRA 3, TESCAN Brno, s.r.o., Brno-Kohoutovice, Czech Republic) image revealed the distinctive dodecahedral and octahedron structures of the Zr^4+^-NMOFs. Subsequently, the bipyridine structure in Zr^4+^-NMOFs facilitated chelation with Fe^3+^ ions. To achieve this, FeCl_3_ was added to the Zr^4+^-NMOFs solution, leading to the formation of the Fe^3+^-NMOFs with a uniform distribution of Fe^3+^ ions. Notably, this modification was accompanied by a color change from white to yellow, as visually observed in Figure S1, indicating the successful chelation of Fe^3+^ ions. Next, Fe-N-C nanozymes were obtained by calcinating Fe^3+^-NMOFs under a N_2_ atmosphere. After calcination, despite a certain degree of agglomeration occurring in the calcined material, the majority of the material still retained the original three-dimensional structure of Fe^3+^-NMOFs (Figure 1b). The calcination process resulted in a smooth edge of the polyhedron, an obviously rough surface, and the formation of numerous tiny nanoparticles, possibly due to metal nanoparticle growth during calcination. The TEM (Talos F200S G2, Waltham, MA, USA) image of Fe-N-C nanozyme in Figure 1c further verified the roughness of the skeleton structure. In the magnified view, the material exhibited dense ~4.3 nm (calculation of 100 particles, pointed by the arrow in Figure 1d) tiny nanoparticles scattered throughout (Figure 1d). The presence of these closely distributed nanoparticles is believed to increase the surface-active sites of the nanozyme, potentially enhancing enzyme activity. Furthermore, a high-resolution TEM of Fe-N-C (Figure 1e) clearly shows a well-defined lattice, indicating the material possesses a good crystalline structure. In addition, the element mapping images of Fe-N-C obtained from high-resolution TEM exhibit a uniform distribution of Fe, O, C, N, and Zr elements on the material (Figure 1f), indicating the successful preparation of the Fe-N-C nanozyme.

**Figure 2:**
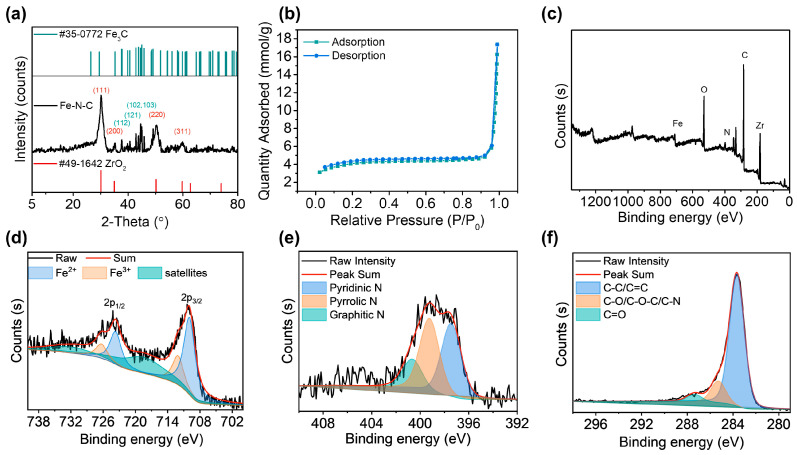
(**a**) The XRD pattern of Fe-N-C nanozyme. (**b**,**c**) Nitrogen adsorption-desorption isotherm and the XPS spectrum of Fe-N-C nanozyme. (**d**–**f**) The high-resolution XPS spectra of Fe 2p, N 1s, and C 1s of the Fe-N-C nanozyme, respectively.

**Figure 3:**
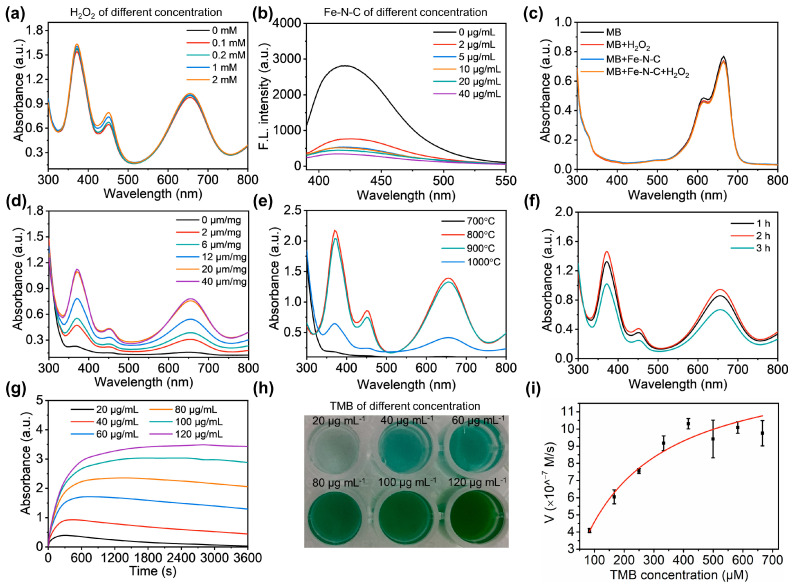
(**a**) UV-vis absorption spectra of TMB solution treated with Fe-N-C nanozymes in the absence or presence of H_2_O_2_. (**b**) Fluorescence spectra of DHE incubated with Fe-N-C nanozyme of different concentrations. (**c**) Absorption spectra of MB treated with different systems. (**d**–**f**) Absorption spectra of TMB solution treated with Fe-N-C nanozyme at different doping amounts of Fe^3+^, calcination temperatures, and calcination times. (**g**) Dynamic monitoring of TMB solution treated with Fe-N-C nanozyme at a series concentration. (**h**) The color change of (**g**) solution. (**i**) The steady-state kinetic assays of oxidation of TMB Fe-N-C nanozyme catalyzed.

The crystallization and phase structure information of Fe-N-C nanozymes were further investigated by XRD (Ultima IV, Rigaku, Japan). As Figure 2a depicts, the crystal spectrum of Fe-N-C nanozyme exhibited a high similarity with the standard Fe_3_C (PDF # 35-0772, lattice parameters, a = 5.091 Å, b = 6.7434 Å, c = 4.526 Å, a = b = c = 90°) with the peaks at 30.1°, 35.2°, 50.4°, and 59.8° corresponding to the lattice fringes of (111), (200), (220), and (311), respectively, and ZrO_2_ (PDF # 49-1642, a = b = c = 5.128 Å, a = b = c = 90°) with the peaks at 37.6°, 42.8°, 44.6°, and 44.9° corresponding to the lattice fringes of (112), (121), (102) and (103), respectively. These results provided evidence of the presence of Fe_x_C and ZrO_2_ in the Fe-N-C nanozyme. Figure 2b provides the N_2_ adsorption and desorption curves of Fe-N-C nanozymes. The specific surface area of Fe-N-C nanozyme was calculated to be 300.04 m^2^ g^−1^. Indeed, the appropriate specific surface area enables the Fe-N-C nanozyme not only to possess highly reactive sites but also to facilitate the interaction of reactants with its structure. In addition, the presence of C, N, O, Fe, and Zr elements in the Fe-N-C nanozyme was further confirmed through XPS analysis (Thermo Fischer, Waltham, MA, USA) (Figure 2c). As shown in Figure 2d, the high-resolution XPS spectra of Fe 2p orbits revealed a pair of bimodal signals corresponding to Fe^3+^ (726.3 eV and 712.2 eV) and Fe^2+^ (723.8 eV and 710.0 eV), which were attributed to the Fe 2p_1/2_ and Fe 2p_3/2_, respectively. This finding indicated the coexistence of both Fe^3+^ and Fe^2+^ in the Fe-N-C nanozyme [31]. The signals at 733.1 eV and 719.5 eV might be the metallic vibration satellite signals. Simultaneously, the high-resolution XPS spectra of Fe 2p revealed the absence of Fe (0) [31,32,33]. Meanwhile, the high-resolution N 1s spectra showed the presence of pyridine N, pyrrolidine N, and graphite N in the Fe-N-C nanozyme (Figure 2e). Previous studies have shown that pyridine N could enhance O_2_ reduction by increasing the π state density, current density, and spin density of C atoms near the Fermi level [31,32,33,34,35]. Moreover, the presence of a pair of lone electrons in the pyridine N structure facilitated the adsorption of reduced O_2_. Therefore, the substantial proportion of pyridine N (~41.6%) in Fe-N-C significantly accelerated the redox reaction, thereby improving the enzyme-like activity of the nanomaterial [33,34,35]. Furthermore, the high-resolution C 1s spectrum (Figure 3f) revealed three characteristic peaks at 287.4, 285.3, and 283.7 eV, corresponding to the C=O, C-O/C-O-C/C-N, and C-C/C=C bonds, respectively [36].

### 3.2. Verification and Optimization of the Enzymatic Activity of Fe-N-C Nanozyme

In previous studies, the Fe-N-C nanomaterials often displayed POD-like activity [24,26]. Thus, in our preliminary experiment, we used the TMB oxidation experiments to test the POD-like enzymatic activity of the Fe-N-C nanoparticles. These experiments involved the oxidation of TMB to a blue-colored product, TMB_ox_ (λ = 652 nm), in the presence or absence of H_2_O_2_ and Fe-N-C nanozymes. Surprisingly, we found that the Fe-N-C nanozyme could catalyze the oxidation of the TMB in the absence of H_2_O_2_ (Figure 3a). This finding indicated that the Fe-N-C nanozyme exhibited OXD-like activity. Meanwhile, Figure 3a demonstrated that the addition of H_2_O_2_ had no significant effect on the catalyzed oxidation of TMB by Fe-N-C nanozyme, indicating that the nanomaterial did not have POD-like activity. In addition, our attempts to eliminate ZrCl_4_ from the Fe-N-C nanozyme through immersion in a sulfuric acid/ammonium sulfate solution were successful. However, the results showed a substantial decline in the catalytic oxidase activity of the treated substances (Figure S2). This decline could possibly be attributed to sulfuric acid’s capacity to also extract a portion of the Fe element. Therefore, Zr content remained in the Fe-N-C nanozyme. To confirm the enzymatic activity, we measured the free radicals produced within the systems. Particularly, the probes DHE, MB, and TA were employed to detect O_2_^•−^ and OH, respectively. The O_2_^•−^ can dehydrogenate the DHE to form the red product, ethidium bromide (Figure S3), leading to a decrease in the fluorescence signals of the DHE [28,37]. Thus, Fe-N-C nanozymes of different concentrations were added to the DHE solution while monitoring the fluorescence variation. Figure 3b showed a significant change in the fluorescence of DHE at 417 nm in the presence of Fe-N-C nanozyme, with the fluorescence intensity decreasing as the material concentration increased. These results demonstrate the generation of the O_2_^•−^ in the catalyzed system, and the Fe-N-C nanozyme exhibited OXD-like activity. In contrast, in the presence of H_2_O_2_, the Fe-N-C nanozyme demonstrated no capability to degrade MB (no change in the color of the solution) or oxidize TA to produce the fluorescent product, 2-hydroxyterephthalic acid (Figure 3c and Figure S4). This observation suggested that •OH was not generated in the reaction system, indicating that the Fe-N-C nanozyme did not exhibit POD-like activity.

Since we have verified that our MOF-derived Fe-N-C nanozyme possesses OXD-mimic activity, we studied the optimum synthesis conditions for the preparation of Fe-N-C nanozyme, including the doping amount of Fe^3+^ ions, calcination temperature, and calcination time. Firstly, a series of Fe^3+^ ions ranging from 0 to 40 μmol mg^−1^ were chelated to the Zr^4+^-NMOFs for the synthesis of the Fe-N-C nanozyme. Despite using different Fe^3+^ ions in Fe-N-C no obvious differences were observed in the resulting Fe-N-C nanoparticle’s three-dimensional structures as characterized by SEM (Figure S5I–VI). However, the catalyzed oxidation experiment using TMB revealed that Fe-N-C nanoparticles without Fe^3+^ ion modification (donated as Fe_0_-N-C) displayed almost no OXD-mimic activity (Figure 3d). As the content of Fe^3+^ ions increased, the TMB oxidation reaction catalyzed by Fe_x_-N-C gradually enhanced, indicating that the OXD-mimic activity sites were primarily provided by Fe elements. Upon reaching a modified concentration of 20 μmol mg^−1^ Fe^3+^ ions, the OXD-mimic activity of prepared Fe-N-C nanozymes showed no further enhancement, suggesting that Fe modification reached saturation at 20 μmol mg^−1^. Next, the calcination temperature and calcination time were investigated, respectively. The results manifested that the OXD-mimic activity of the Fe-N-C nanozyme was comparable at 700 °C, 800 °C, and 900 °C (donated as Fe-N-C_700_, Fe-N-C_800_, and Fe-N-C_900_), with the Fe-N-C_800_ nanozyme exerting relatively slightly better performance (Figure 3e). However, when the calcination temperature reached 1000 °C, the OXD-mimic activity of the Fe-N-C_1000_ decreased sharply. The morphologies of Fe-N-C nanozyme calcination at different temperatures are shown in Figure S6I–IV. At a calcination temperature of 700 °C, the Fe-N-C_700_ nanoparticles were not fully calcined, and some nanoparticles still exhibited smooth surfaces. Fe-N-C_800_ nanozyme and Fe-N-C_900_ nanozyme exhibited well-defined structures. However, at a calcination temperature of 1000 °C, the material experienced obvious agglomeration, which might be the main reason for the deterioration of oxidase performance. Consequently, 800 °C was selected as the final calcination temperature. Next, calcination time was also optimized (Figure 3f and Figure S7I–III), and 2 h showed the best performance. Thus, the Fe-N-C nanozyme was obtained by calcinating at 800 °C for 2 h. In addition, the OXD-mimic activity of Fe-N-C nanozymes was systematically investigated through the TMB OXD experiment. As the concentration of Fe-N-C nanozyme increased, the efficiency of TMB oxidation was gradually enhanced (Figure 3g), leading to a deepening of the blue color of the oxidized product (Figure 3h). In order to determine the reaction rate values, catalytic experiments of Fe-N-C nanozyme on TMB with varying concentrations were performed. By fitting the Michaelis–Menten equation, we obtained the *K_m_* and *V_m_* constants of 2.1 × 10^−4^ M and 1.4 × 10^−6^ M s^−1^, respectively (Figure 3i).

### 3.3. Detection of ALP by the Fe-N-C Nanozyme Based System

**Figure 4:**
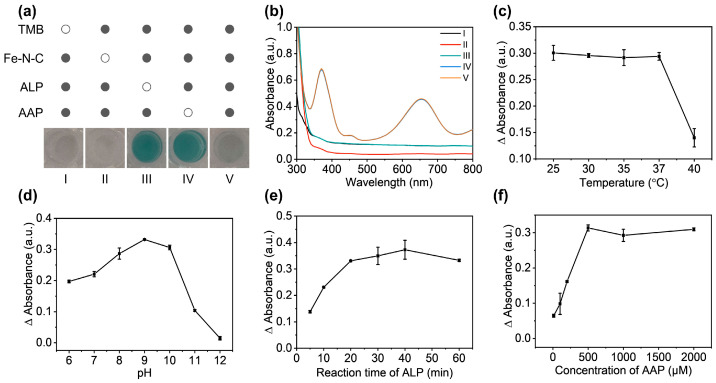
(**a**) The feasibility of the Fe-N-C nanozyme-based system for ALP detection. The white dots and grey dots represented the absence and presence of the corresponding substance, respectively. I: Fe-N-C nanozyme + ALP + AAP; II: TMB + ALP + AAP; III: TMB + Fe-N-C nanozyme + AAP; IV: TMB + Fe-N-C nanozyme + ALP; V: TMB + Fe-N-C nanozyme + ALP + AAP. (**b**) The UV-vis absorption spectra of TMB oxidation under different conditions correspond to (**a**). (**c**–**f**) The optimization of the experimental conditions for ALP detections included the incubation temperature, pH, reaction time, and concentration of AAP, respectively.

The principle for ALP detection is depicted in Scheme 1b. Initially, the ALP catalyzed the dephosphorylation of the substrate, AAP, leading to the generation of the reductive product, AA. The presence of AA significantly inhibited the OXD-mimic activity of Fe-N-C nanozyme and weakened the catalytic oxidation of TMB to produce the TMB_ox_ (Figure 4a). Thus, in the presence of the ALP, the oxidation of the TMB by the Fe-N-C nanozyme was suppressed, causing the solution to become colorless. Conversely, in the absence of the ALP, the enzymatic dephosphorylation process could not happen, leading to the absence of AA formation. Under this condition, the Fe-N-C nanozyme could oxidize the TMB, generating the blue-colored product, TMB_ox_. In order to make the visualization clear, rhodamine was introduced into the detection system as a reference substance.

In order to verify the potential of Fe-N-C nanozymes for detecting ALP, a feasibility analysis of the method was performed. As shown in Figure 4a,b, in the absence of TMB or Fe-N-C, no blue TMB_ox_ was generated in the reaction system. However, in the presence of both TMB and Fe-N-C nanozymes, they coexisted without ALP or AAP in the solution, leading to the catalysis of TMB into TMB_ox_. Conversely, when TMB, Fe-N-C, ALP, and AAP were present together, the OXD property of Fe-N-C was inhibited, resulting in a colorless solution. Therefore, the difference in the absorbance value of the reaction system with or without ALP and the dependence of ALP concentration allowed for the rapid detection of ALP by using the Fe-N-C nanozyme.

In the detection system, we defined the absorbance value of the Fe-N-C nanozyme-catalyzed oxidation of TMB in the absence of ALP as A_0_, while the value in the presence of ALP was regarded as A. Thus, the A_0_ − A (ΔA) value could reflect the detection signal of ALP. In order to obtain the optimal experiment conditions, the temperature, pH value, and reaction time of ALP for detection were investigated. As shown in Figure 4c, ΔA values for the ALP detection remained consistent in the range of temperatures from 25 °C to 37 °C. However, when the temperature surpassed 40 °C, the ΔA decreased sharply, indicating a significant reduction in the enzymatic activity of ALP (Figure 4c). In order to obtain excellent performance, 37 °C was selected for the subsequent experiments. In addition, as for pHs, the detection sensitivity of ALP gradually increased within the range of pH 6~9, and the optimal pH condition was achieved at pH 9. Further increasing the pH from 10~12, the ΔA values dropped sharply, indicating the inhibition of ALP enzymatic activity under strong alkali conditions (Figure 4d).

Next, the optimal reaction time of ALP and the concentration of AAP were investigated. It was observed that the catalytic reaction rate increased rapidly within 20 min and reached its highest value at 40 min (Figure 4e). Moreover, the result revealed that the substrate concentration for ALP detection showed a similar trend, and 500 μM of AAP was selected for the subsequent experiments (Figure 4f). In summary, the optimum conditions for ALP detection were 500 μM AAP in Tris-HCl buffer (pH 9) at 37 °C with an incubation time of 40 min. In addition, we optimized the volume ratio of the ALP mixture and TMB solution (Figure S8). When the volume ratio was lower than 5:5, the ΔA was low due to the limited content of AA in the system. Conversely, when the volume ratio was higher than 5:5, the TMB catalytic system under an acidic pH environment was disrupted, leading to the inhibition of the oxidization reaction and a significant reduction in ΔA. As a result, the volume ratio of 5:5 was chosen for further experiments.

**Figure 5:**
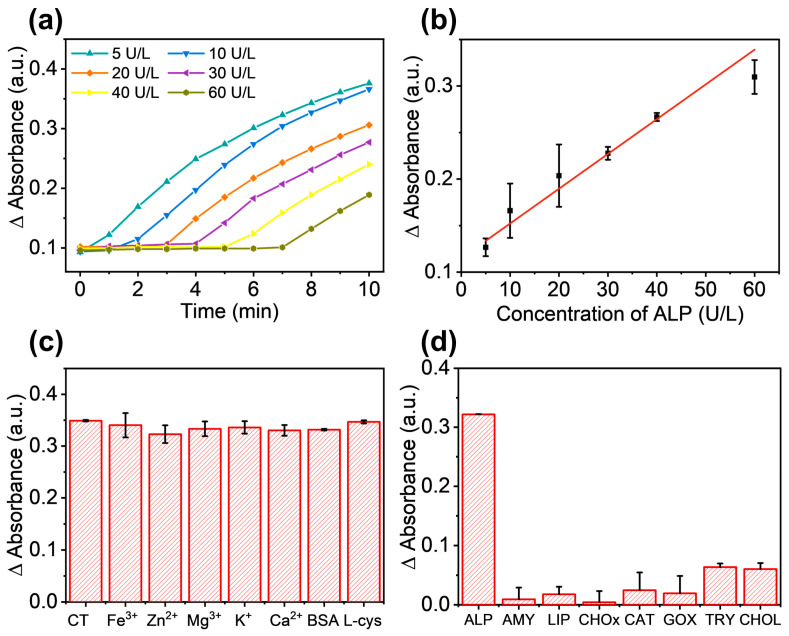
(**a**) Dynamic monitoring of ALP at different concentrations treated with Fe-N-C nanozyme. (**b**) Linear relationship between concentrations of ALP and ΔA at 652 nm. (**c**) Evaluation of the anti-interference capability of Fe-N-C-based system for ALP detection. (**d**) Verification of the specificity of our method for ALP detection.

Finally, we performed the ALP detection under optimal conditions. Particularly, the absorbance value of mixed solutions of ALP and TMB was continuously monitored at 652 nm (Figure 5a). The results showed that as the concentration of ALP increased, the inhibition time and effect of TMB oxidation by AA also increased. The inhibition effect reached a stable state at 10 min. Therefore, we chose 10 min as the catalytic duration for TMB oxidation. Next, the ΔA at 652 nm for ALP concentrations in the range of 5 to 60 U L^−1^ was recorded, and the linear fitting was performed in Figure 5b. The linear regression equation was ΔA_652 nm_ = 3.74 × 10^−3^ C_ALP_ + 0.11 (U L^−1^, R^2^ = 0.98), and the LOD was calculated to be 3.38 U L^−1^. In addition, the calculated recoveries of ALP (5, 30, 40 U L^−1^) were 99.23 ± 12.12%, 100.52 ± 6.17%, and 101.46 ± 2.91%, exhibiting the well-practicability of the Fe-N-C nanozyme for ALP detection (Table S1).

In addition, to evaluate the anti-interference performance of our method in ALP detection, 0.01 mM of Fe^3+^, Zn^2+^, Mg^2+^, K^+^, Ca^2+^, BSA, and L-cys were individually added to the ALP detection system. The results showed that ΔA values of all samples remained stable (Figure 5c), indicating the good anti-interference performance of this method. Next, we used AMY, LIP, CHOx, CAT, GOX, TRY, and CHOL as control groups to verify the specificity of the system. As depicted in Figure 5d, the hydrolysis impacts of these seven control enzymes on AAP were minimal, resulting in small ΔA values. Therefore, the Fe-N-C nanozyme-based system showed good anti-interference properties and selectivity for ALP detection.

### 3.4. Detection of ALP in Clinical Samples

**Figure 6:**
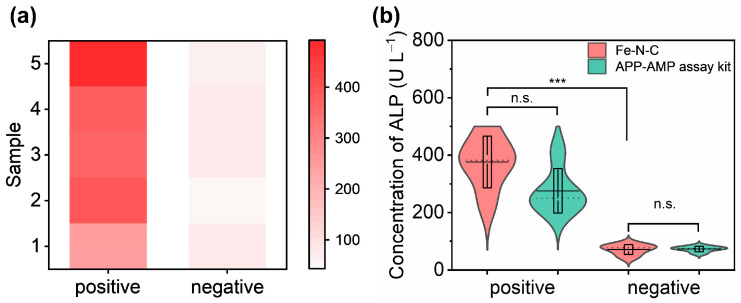
(**a**) The heatmap of detection of ALP in the clinical samples. (**b**) The box and violin image for comparing the measured values by the Fe-N-C nanozyme-based system and the APP-AMP assay kit. (*** *p* < 0.001, n.s. represented no significance.).

In order to evaluate its practical application, we used our Fe-N-C nanozyme-based method to detect ALP in clinical samples. A total of 10 serum samples were obtained from Renming Hospital of Wuhan University, including 5 from healthy donors and 5 from patients with abnormal conditions. The results for the ALP detection were shown in Figure 6a,b. As shown in Figure 6a,b, there was no significant difference between the measured values obtained by using the Fe-N-C nanozyme-based system and those obtained by the AAP-AMP assay kits, which were provided by the hospital. In addition, the value of negative samples measured by the Fe-N-C nanozyme-based system showed significant differences from positive samples. These findings confirmed the successful application of the Fe-N-C-based ALP detection method for rapid quantitative analysis of ALP in clinical samples. Although our Fe-N-C-based method demonstrated outstanding potential for real-sample testing, extensive statistical cohorts should be recruited, and further experiments are required for validation.

### 3.5. Visual Detection of ALP

**Figure 7:**
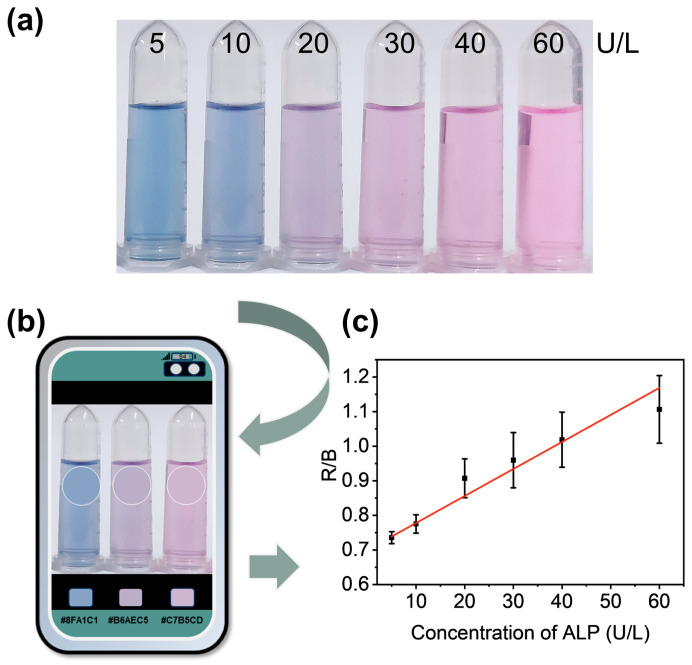
(**a**) The color changes under different concentrations of ALP after the addition of Rhodamine (Color Collect APP, 72 dpi). (**b**) Schematic representation of RGB values read by a smartphone. (**c**) Linear relationship between R/B value and the concentration of ALP.

Considering the color change during TMB catalytic oxidation, we introduced rhodamine to the solution as a reference substance, enabling the development of a visual detection method by using a smartphone APP. This method could achieve POCT for ALP, offering the advantages of low cost, rapid operation, and simplicity. Figure 7 shows the color changes of the solution under different concentrations of ALP (Color Collect APP, 72 dpi). The operation procedure is shown in Figure S9. As the concentration of ALP increased, the color of the solution gradually changed from blue to purple, eventually turning pink for visual detection. By reading the RGB value of the image using a smartphone, we used the R/B values as the ordinate to fit the linear regression equation as I_R/B_ = 7.82 × 10^−3^ C_ALP_ + 0.70 (U L^−1^, R^2^ = 0.95, LOD = 2.12 U L^−1^). Compared with literature reports (Table 1), our method showed comparable sensitivity and a similar linear range. The results confirmed that the colorimetric analysis of our method also displayed a similar linear range and a satisfactory LOD when compared with the ΔA system.

## 4. Conclusions

In conclusion, we successfully synthesized the UiO-67 MOF-derived Fe-N-C nanozyme, which exhibited exceptional oxidase-mimic catalytic activity, enabling efficient oxidation of TMB without the requirement of H_2_O_2_. By incorporating the ALP enzymatic catalytic reaction to inhibit the oxidation of TMB by Fe-N-C nanozyme, we developed a rapid and highly sensitive approach for the detection of ALP under optimal conditions. The approach successfully realized sensitive detection of ALP in both buffer and clinical samples by using the colorimetric assay. Moreover, we introduced a visual detection method by using a smartphone-based colorimetric assay, facilitating practical and accessible POCT for ALP, even in resource-limited settings. Overall, we constructed a new UiO-67 MOF-derived Fe-N-C nanozyme, and the utilization of the Fe-N-C oxidase nanozyme and the visual detection method holds significant implications for advancing point-of-care diagnostics in diverse healthcare settings, contributing to the progress of rapid and accurate diagnostic technologies for improved patient care.

## Figures and Tables

**Table 1:** Comparison of detection method of ALP.

Material	Method	Liner Range (U L^−1^)	LOD (U L^−1^)	Detection Time	Ref.
TPE-CN-pho ^a^	Fluorescence	25~175	14.2	60 min	[36]
P/DS/EDC-NHS/Anti-ALP biosensor ^b^	Visual detection	10^4^~10^6^	870	13 min	[6]
HRP-TMB-H_2_O_2_ with the Cu^2+^ system	UV spectrum	0–120	5.4	60 min	[38]
CsPbBr_3_ NC	Photocurrent responses	50~1000	42.1	30 min	[39]
CsPbBr_3_@PMMA	Fluorescence	10~100	4.8	70 min	[40]
CuNPs-Cr^3+^-PPi	Fluorescence	0~62.5	3.3	30 min	[41]
Fe-N-C	UV spectrum/Visual detection	5~60	3.38/2.12	50 min	This work

^a^: tetraphenylenthene-cyano-phosphate group; ^b^: Whatman filter paper/4-carboxybenzene diazonium/EDC-NHS/Anti-ALP biosensor.

## Data Availability

The research data presented in this study are available on request from the corresponding author.

## Supplementary Materials

The following supporting information can be downloaded at: https://www.mdpi.com/article/10.3390/nano13182496/s1, Figure S1: The optical pictures of (I) Zr^4+^-NMOFs, (II) Fe^3+^-NMOFs, and (III) Fe-N-C; Figure S2: The oxidase activity of the Fe-N-C (left) and treated with sulfuric acid/ammonium sulfate solution (right). The TMB oxidation reaction was used to monitor the oxidase activity. Figure S3: The optical pictures of DHE incubated with different concentrations of Fe-N-C. From left to right were 0, 2, 5, 10, 20, and 40 μg mL^−1^, respectively; Figure S4: Fluorescence spectra of TA incubated with different systems; Figure S5: The SEM images of Fe-N-C with different content of Fe elements; Figure S6: The SEM images of Fe-N-C obtained at different calcination temperatures; Figure S7: The SEM images of Fe-N-C obtained by different calcination times; Figure S8: The ΔA of the Fe-N-C system treated with different volume ratios of ALP and TMB solutions. Figure S9: Procedure for the Color Collect APP to read the RGB value of the images. Table S1: Recovery of ALP activity determined by Fe-N-C nanozyme.
