# Supplementary material for: Construction of Metal Organic Framework-Derived Fe-N-C Oxidase Nanozyme for Rapid and Sensitive Detection of Alkaline Phosphatase
Source: Nanomaterials (Basel). 2023 Sep 5;13(18):2496. doi: 10.3390/nano13182496 (PMC10537703; doi:10.3390/nano13182496)
Supplement: Supplementary file 1 [file nanomaterials-13-02496-s001.zip › nanomaterials-2561073-supplementary.pdf]

nanomaterials

Supplementary Materials

Construction of Metal Organic Framework-Derived Fe-N-C Oxidase Nanozyme for Rapid and Sensitive Detection of Alkaline Phosphatase

Mengmeng Pan [1], Ming Wang [2], Linjiao Yang [1], Yongli Song [1], Ming Jiang [1], Xu Yu [1,3,*] and Li Xu [1,3,*]

[1] Tongji School of Pharmacy, Huazhong University of Science and Technology, Wuhan 430030, China; d202081590@hust.edu.cn (M.P.); d202081597@hust.edu.cn (Y.S.); 2001020636@hust.edu.cn (M.J.)
[2] Department of Clinical Laboratory, Renmin Hospital of Wuhan University, Wuhan 430060, China; morgan@whu.edu.cn
[3] Hubei Jiangxia Laboratory, Wuhan 430200, China
* Correspondence: xuyu@hust.edu.cn (X.Y.); xulpharm@mails.tjmu.edu.cn (L.X.)

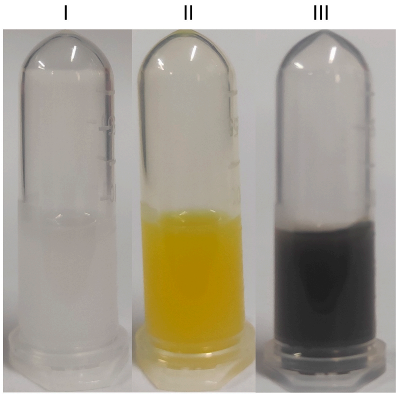

Figure S1. The optical pictures of (I) Zr^{4+}-NMOFs, (II) Fe^{3+}-NMOFs and (III) Fe-N-C.

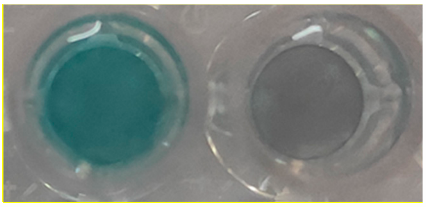

Figure S2. The oxidase activity of the Fe-N-C (left) and treated with sulfuric acid/ammonium sulfate solution (right). The TMB oxidation reaction was used to monitor the oxidase activity.

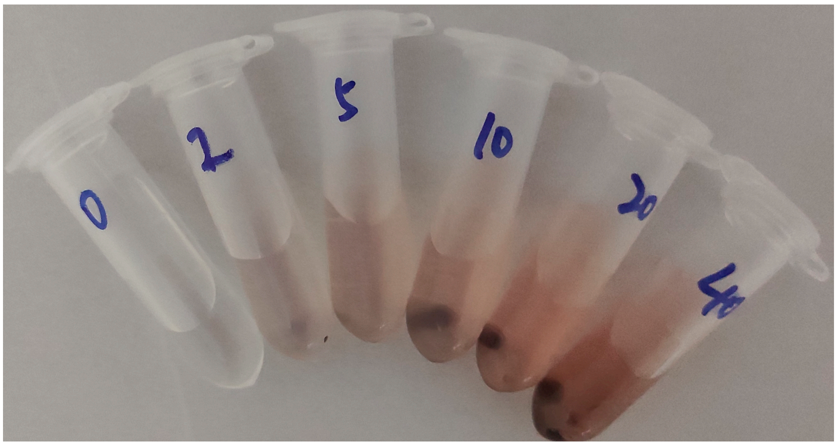

Figure S3. The optical pictures of DHE incubated with different concentration of Fe-N-C. From left to right were 0, 2, 5, 10, 20, 40 μg mL^{-1} respectively.

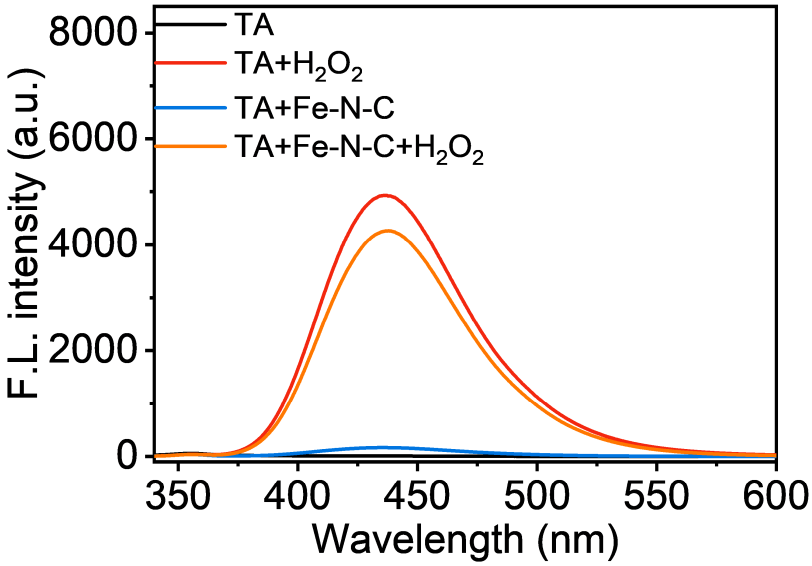

Figure S4. Fluorescence spectra of TA incubated with different system.

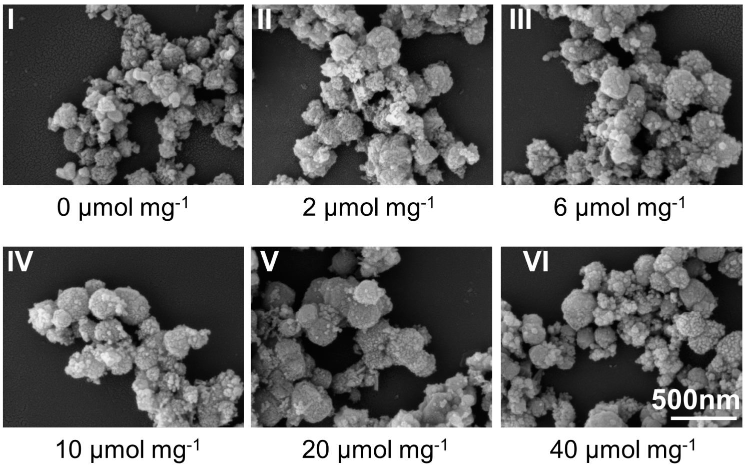

Figure S5. The SEM images of Fe-N-C with different content of Fe element.

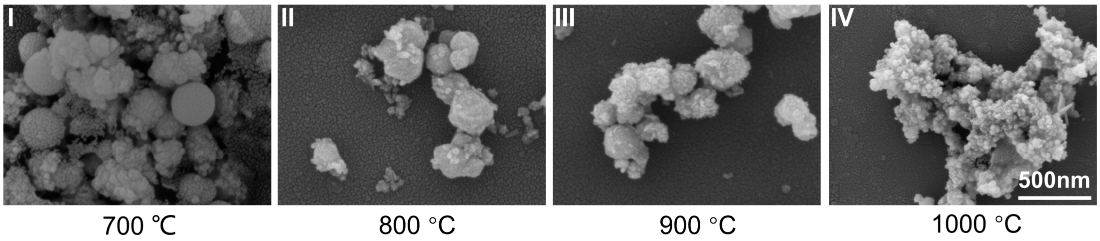

Figure S6. The SEM images of Fe-N-C obtained by different calcination temperatures.

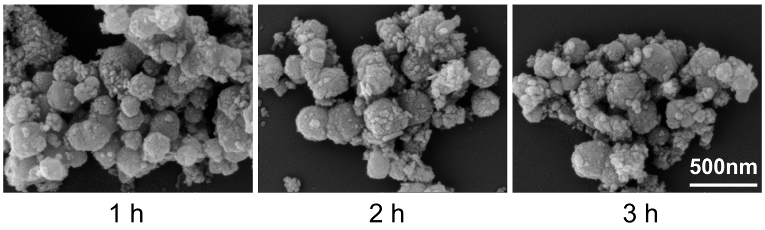

Figure S7. The SEM images of Fe-N-C obtained by different calcination times.

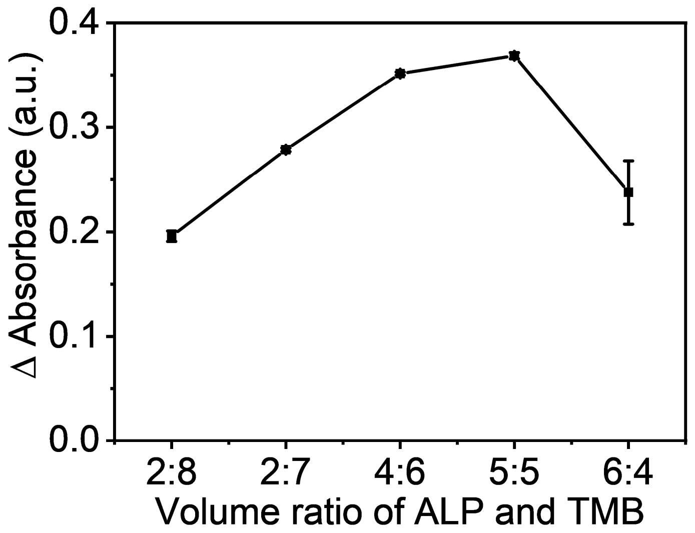

Figure S8. The ΔA of Fe-N-C system treated with different volume ratio of ALP and TMB solution.

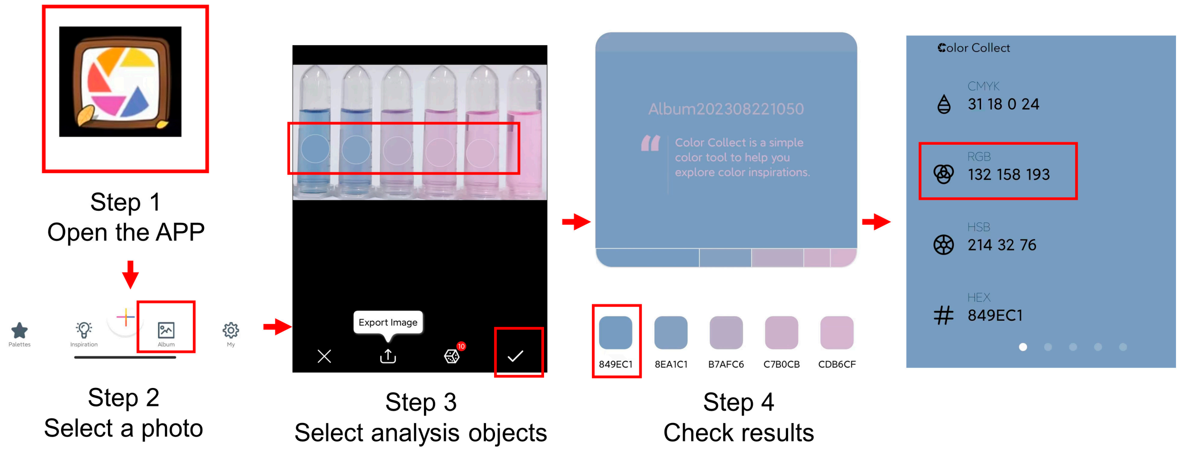

Figure S9. Procedure for the Color Collect APP to read the RGB value of the images.

Table S1 Recovery of ALP activity determined by Fe-N-C nanozyme

Sample	Added (U L^{-1})	Fe-N-C assay (U L^{-1})	Recovery (%)	SD (%, n=3)
1	5.00	4.96	99.23	12.12
2	30.00	30.16	100.52	6.17
3	40.00	40.58	101.46	2.91
